# Circadian Sleep-Activity Rhythm across Ages in Down Syndrome

**DOI:** 10.3390/brainsci11111403

**Published:** 2021-10-25

**Authors:** Annalysa Lovos, Kenneth Bottrill, Stella Sakhon, Casandra Nyhuis, Elizabeth Egleson, Alison Luongo, Melanie Murphy, Angela John Thurman, Leonard Abbeduto, Nancy Raitano Lee, Katharine Hughes, Jamie Edgin

**Affiliations:** 1 Department of Psychology, School of Mind, Brain and Behavior, College of Science, The University of Arizona, Tucson, AZ 85721, USA; kbottrill@email.arizona.edu (K.B.); egleson@email.arizona.edu (E.E.); alisonluongo@email.arizona.edu (A.L.); jedgin@email.arizona.edu (J.E.); 2 Statistics Department, Los Angeles Valley College, Van Nuys, Los Angeles, CA 91401, USA; ssakhon0106@gmail.com; 3 College of Medicine, Pennsylvania State University, Hershey, PA 17033, USA; cnyhuis1@jhmi.edu; 4 Department of Physiology and Buiphysics, School of Medicine, Case Western Reserve University, Cleveland, OH 44106, USA; mxm1375@case.edu; 5 Department of Psychiatry and Behavioral Sciences and MIND Institute, University of California Davis Health, Sacramento, CA 95817, USA; ajthurman@ucdavis.edu (A.J.T.); ljabbeduto@ucdavis.edu (L.A.); 6 Department of Psychological and Brain Sciences, Drexel University, Philadelphia, PA 19104, USA; nrl39@drexel.edu; 7 Goldsmiths College, University of London, London SE14 6NW, UK; kmohughes.89@gmail.com; 8 Sonoran University Center for Excellence in Developmental Disabilities (UCEDD), University of Arizona, Farmington, CT 06032, USA

**Keywords:** Down syndrome, sleep, circadian rhythms, memory, executive function, reaction time, brain development

## Abstract

Across all ages, individuals with Down syndrome (DS) experience high rates of sleep problems as well as cognitive impairments. This study sought to investigate whether circadian rhythm disruption was also experienced by people with DS and whether this kind of sleep disorder may be correlated with cognitive performance. A cross-sectional study of 101 participants (58 with DS, 43 with typical development) included individuals in middle childhood (6–10 years old), adolescence (11–18 years old), and young adulthood (19–26 years old). Sleep and markers of circadian timing and robustness were calculated using actigraphy. Cognitive and behavioral data were gathered via a novel touchscreen battery (A-MAP^TM^, Arizona Memory Assessment for Preschoolers and Special Populations) and parent questionnaire. Results indicated that children and adolescents with DS slept the same amount as peers with typical development, but significant group differences were seen in phase timing. The circadian robustness markers, interdaily stability and intradaily variability of sleep-wake rhythms, were healthiest for children regardless of diagnostic group and worst for adults with DS. Amplitude of the 24-h activity profile was elevated for all individuals with DS. In analyses of the correlations between sleep quality, rhythms, and cognition in people with DS, interdaily stability was positively correlated with reaction time and negatively correlated with verbal and scene recall, a finding that indicates increased stability may paradoxically correlate with poorer cognitive outcomes. Further, we found no relations with sleep efficiency previously found in preschool and adult samples. Therefore, the current findings suggest that a thorough examination of sleep disorders in DS must take into account age as well as circadian robustness to better understand sleep-cognitive correlations in this group.

## 1. Introduction

Very little is known about the rhythmicity of sleep-wake cycles in individuals with Down syndrome (DS), even though high rates of other sleep disorders are well documented in this population. Sleep fragmentation, obstructive sleep apnea, and low sleep efficiency have been the most frequently noted sleep disorders and are well documented for infants [1], children [2,3], and adults with DS [4]. Occurrence rates in this population have been estimated to be as high as 31–66% for sleep-disordered breathing and 52–69% for behavioral sleep problems [5,6].

Individuals with DS display a profile of relative strengths [7] and challenges; the latter including both physical health and cognitive features. One of the physical features common to individuals with DS is mid-facial hyperplasia and macroglossia, which contribute to reduced upper airway dimensions [8,9]. This together with the hypotonia common in people with DS (which can contribute to upper airway collapse during sleep), yields conditions in which sleep-disordered breathing often occurs. Sleep-disordered breathing, in turn, is a primary cause of fragmented sleep and can be responsible for reduced total sleep time, poor sleep quality and daytime sleepiness, as well as behavioral problems and cognitive limitations [3,10]. Such cognitive effects due to sleep difficulties could compound the mild to moderate existing cognitive deficits already experienced by individuals with DS. 

The urge to sleep represents a fundamental physiological drive that is essential for the growth and development of the body and brain during childhood and adolescence, and for ongoing repair and maintenance processes throughout the lifespan. Sleep is also thought to be of fundamental importance for learning in allowing for “offline” integration and consolidation processes of memory [11]. Disrupted sleep is especially worrisome during childhood and adolescence, when the central nervous system is still developing and therefore vulnerable to insults that could have both short-term and long-term effects [12,13].

Some of the consequences of sleep disturbance that have been noted in the research on young people with DS are daytime behavior issues and parent-reported executive function deficits [14]; lower verbal intelligence and poorer cognitive flexibility [3]; and reduced communicative skills [15]. Furthermore, a recent structural MRI study of youth with DS by Lee et al. offers preliminary indications that disturbed sleep in these youth could affect the very structure of the developing brain. As Lee et al. report, frontal, temporal and parietal total volumes were significantly reduced in youth with DS who were also poor sleepers when compared to both neurotypical controls and youth with DS who were not reported to demonstrate disturbed sleep [16]. 

Although sleep problems in individuals with DS and their cognitive associations are well documented, the same is not true for circadian cycles of sleep and activity in this population. Studies of rhythmicity in neurotypical humans show that circadian rhythms are endogenous; they occur even in conditions of constant darkness [17]. Neurotypical adults also have rhythms that align closely with (but are often slightly longer than) a 24-h period [17]. Through entrainment, we are typically able to synchronize our sleep and wake activity with the environment using zeitgebers (external cues which entrain an organism’s biological rhythms) like light, temperature, and eating and drinking patterns. 

The elements necessary for human circadian structure are present at birth but come together in the weeks and months that follow. In an in-home longitudinal study of circadian development in infants with typical development (TD), Joseph et al. measured sleep and circadian markers, and found that cortisol levels stabilized into a circadian rhythm first at about 8 weeks of age, followed by melatonin at about 9 weeks of age. Sleep efficiency stabilized next, at about 10 weeks of age, followed by core body temperature and circadian gene expression at about 11 weeks of age [18]. It thus appears that in neurotypical humans, biological rhythms are established early in life, led by cortisol secretion, and associated with the development of more consolidated nighttime sleeping.

The consolidation of 24-h rhythms into consistent periods of sleep and wake is an important step that has implications for physical and mental health. Converging results from several studies indicate that circadian rhythm systems influence cognitive function independently of sleep [19,20]. In adolescents with TD, circadian rhythm disruption has been linked to excessive sleepiness, impaired mood regulation, school tardiness and absenteeism, higher rates of substance use, and lower high school graduation rates [21,22,23]. In neurotypical adults, one forced desynchrony study found that participants with circadian rhythm disruption as well as sleep impairment showed further learning impairments (working and long-term memory, counting, reaction time, retrieval speed of number facts and verbal information) beyond the cognitive impairment found in a group that was only sleep-impaired, further strengthening the claim that circadian disruption is an independent contributor to cognitive impairment [19]. Other cognitive deficits noted in neurotypical adult studies of circadian disruption include poorer psychomotor performance [24,25,26], poorer general executive function, [24,27], longer visual reaction time [25,28], and decreased accuracy [29]. Given that verbal function, memory, and executive control are challenges in DS, examining the impact of sleep and daily rhythms on these outcomes is an important next step. 

Recent research has also documented a connection between circadian rhythm disruption and Alzheimer’s dementia (AD). As a high percentage of adults with DS develop AD at a relatively young age [30], this connection is particularly relevant. Adults with DS are known to show accelerated aging [31,32], which includes both earlier onset of AD and increased incidence. By the age of 40, nearly all adults with DS demonstrate the characteristic amyloid-β plaques and neurofibrillary tangles present in AD [31,33]. Several recent studies have noted fragmented sleep rhythms and delayed phase in neurotypical individuals with AD [34,35] and have proposed that circadian rhythm disruption may be not only a symptom of neurodegeneration but might also be a potential risk factor for the development of AD [34]. Therefore, further study of sleep-wake cycles across a wider span of ages in adolescents and adults with DS may help researchers better understand the interplay between sleep-wake rhythms and cognition in this population.

There is thus ample evidence that sleep and circadian patterning must both be considered in an examination of sleep health and correlated cognitive outcomes. Furthermore, the development of these states must also be taken into account, as sleep quality and daily rhythms fluctuate with development and age. For instance, it has been widely observed that adolescents tend to exhibit a distinct preference for later phase timing (i.e., later bedtimes and waking times) compared to children, which was until recently attributed to psychosocial factors such as increased responsibility, greater amounts of homework, nighttime socializing with friends and other such pastimes. It has also been found under laboratory conditions, when all psychosocial pressures were removed, that the adolescent preference for later bedtimes and wake times was correlated only with the stage of pubertal development [36]. Thus, the adolescent phase shift can be seen as a maturational process that occurs alongside other biological changes with the onset of puberty. 

Only one study has investigated rhythmicity in the timing of sleeping, waking, and activity levels over a 24-h period in humans with DS; and the study included only infants and young children. In that study, healthy circadian rhythm development was found in participants aged 5–60 months [13]. They also found in these children a slight preference for an earlier, or “lark” chronotype (phase-advancement leading to waking up and going to bed earlier). Cognitive and behavioral outcomes were not tested. The “lark” preference has also been noted in the Ts65Dn mouse model of DS [37], as has robust circadian rhythm development [38]. Considering this tendency to demonstrate phase advancement in studies of animals and human infants with DS, it was anticipated that adolescents with DS might demonstrate a less pronounced adolescent phase shift. However, no one has yet measured phase timing in adolescents with DS.

The current study sought to examine sleep-activity rhythms as markers of phase and circadian rhythmicity in middle childhood, adolescence, and young adulthood in individuals with DS. Using sleep-wake activity profiles gathered via actigraphy as well as sleep diaries, sleep rhythms were characterized for participants (with DS and TD) in middle childhood, adolescence, and early adulthood. We hypothesized that some or all adolescents with DS may begin to show signs of circadian rhythm disruption as measured by interdaily stability (IS), intradaily validity (IV), and amplitude and the FFT (fast Fourier transform) of the amplitude of the 24-h activity cycle; and that furthermore, this disruption might persist into adulthood. For participants with DS, correlations with memory and cognitive function previously found to be related to circadian function in individuals with TD were also examined. We predicted that cognitive measures correlated with sleep fragmentation in previous studies, especially verbal recall and reaction time, may be correlated with circadian rhythm disruption. Finally, we expected to confirm previous findings of group differences between individuals with DS and TD for the measures of total sleep time (TST), sleep efficiency (SE), and awakenings after sleep onset (WASO).

## 2. Materials and Methods

### 2.1. Participants

101 children, adolescents and young adults were included from two studies led by the University of Arizona. Children and adolescents were all enrolled in school or were homeschooled. 41.18% of participants identified as biracial or multiracial, so percentages may add up to greater than 100%. 68.63% of participants identified as White, 36.27% as Hispanic, 7.84% as Asian or Pacific Islander, 4.9% as Black, and 2.4% as American Indian or Alaskan Native. Two participants (1.96%) identified only as other, and 14 (13.7%) provided no specific information. 58 participants had a diagnosis of DS; 43 were age-matched peers with TD. For individuals with DS, genetic diagnosis was confirmed prior to entry into their respective studies. The Kaufman Brief Intelligence Test, version II (KBIT-II) [39] was given to all participants with DS as a measure of global intelligence, which was ≤80 for all with DS. For participants with TD, assignment to the group was made based on absence of developmental delays, absence of prior head trauma, and absence of autism spectrum disorder or anxiety disorder, unless they were on a stable treatment regimen. Additionally, participants in both groups had normal vision (or corrected to normal) and had normal hearing. Individuals with fewer than 5 consecutive full days and nights of actigraphy were not included in the present study; nor were individuals who wore the actiwatch at night only or had taken off the actiwatch for extended periods. No participants wore or began wearing the actiwatch on the date of a daylight savings time transition or within one week following a daylight savings time transition. Demographic information is summarized in Table 1.

### 2.2. Sleep Data

Sleep data were gathered via wrist worn Actiwatch II (Philips Healthcare, Amsterdam, the Netherlands) on 5–7 consecutive days and nights in the home environment. Previous studies have used the Actiwatch II to accurately measure sleep and sleep-wake rhythmicity in children and youth with DS [2,13]. Although not as accurate as polysomnography (PSG) for estimating waking periods during sleep [40], actigraphy is more accessible for studies of children, youth and special populations. It has been found useful in estimating the stability of sleep rhythms [41], correlates well with measurement of the rhythms of melatonin and core body temperature [41] and is well-suited for use in preliminary estimations of circadian phase [42,43].

The Actiwatch II measures activity via an accelerometer and quantifies ambient light levels in lux. Based on these parameters it estimates sleep and wake behavior. Sampling rate was set at 32 Hz with a peak range of sensitivity at 0.5–2 G. All data were collected in 30-s epochs. Parents were instructed to place the Actiwatch II on their child’s non-dominant wrist and to leave it on except for bathing or swimming that lasted longer than 15 min. They were additionally given sleep diaries for recording bedtimes, waking times, sleep periods during the day (naps), and night waking periods for which the child got out of bed. All actigraphy data were scored and double scored according to laboratory standardized procedures that were constant across studies, using Actiware 6.0.9 (Philips Healthcare). Sleep variables quantified via these procedures included total sleep time (TST) in minutes, sleep efficiency (SE), and time awake after sleep onset (WASO) in minutes. Scored actigraphy files containing 2880 data points per each 24-h period were exported to ClockLab 6.0 circadian software (Actimetrics, Wilmette, IL, USA). The data were analyzed as time series to estimate sleep-wake rhythms for each participant. All full 24-h days for each participant were averaged to produce the sleep, phase and circadian robustness estimates on an individual basis.

**Figure 1:**
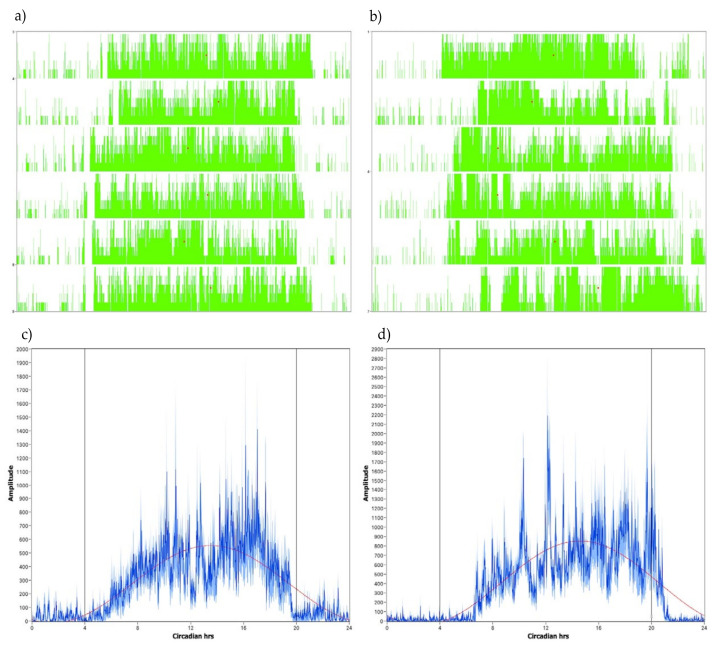
Representative actograms and activity profiles. (**a**) 6-day actogram (1 row per day) of activity counts for a 15-year-old with DS, showing relatively consistent onsets, offsets, and acrophases (acrophases noted with red dots as the midpoint of daily activity). (**b**) 6-day actogram (1 row per day) of activity counts for a 15-year-old with TD, showing a wider range of variation in onsets, offsets and acrophases (noted with red dots). (**c**) 24-h activity profile of 6-year-old with DS, showing the consolidation of waking (higher amplitude) versus sleeping times (lower amplitude). (**d**) 24-h activity profile of a 6-year-old with TD, also showing sleep and wake consolidation.

The phase markers used in this study included daily onsets and daily offsets, calculated as the averaged onset of daily waking and the averaged onset of sleep, respectively. Onsets and offsets were determined using a template-matching algorithm. For onsets, the algorithm searched for a 5-h period of inactivity followed by a 5-h period of activity. For offsets, the algorithm searched for the opposite circumstance: a 5-h period of high activity followed by a 5-h period of relative inactivity. A third phase marker was acrophase, or the average time of day of an individual’s greatest activity. Acrophase was found by fitting each day’s activity to a 24-h sine wave and taking the position of the peak activity [44,45]. Figure 1 displays representative actograms and activity profiles for participants with DS or TD.

The robustness of participants’ sleep-wake rhythms was estimated using several measures, notably the amplitude and the fast-Fourier transform (FFT) of the 24-h activity cycle. The FFT is a standard measure determined using the power spectrum from 18 to 30 h. A measure of MESOR (the midline of the rhythm cycle fit to a sine wave) was also generated. Five measures of Non-Parametric Circadian Rhythm Analyses (NPCRA) were additionally computed to assess robustness of the sleep-wake activity following the formulas published by Van Sommern et al. [46]. The NPCRA measure of Interdaily Stability (IS) provides an index of how consistently a person’s sleep-activity patterns are maintained across the period of actigraphy data collection and range from 0 (not consistently maintained at all) to 1.0 (perfect consistency in day-to-day rhythms) [44,45,46]. Intradaily Variability (IV) is an index quantifying how frequently transitions occur between low-activity periods and high-activity periods in a 24-h day. Persons with circadian rhythm disorders may demonstrate less consolidated sleep and activity periods, which would result in higher IV values. The index ranges from 0 to 2.0, with a value close to 0 indicating that transitions are few, and with a value closer to 2 indicating that transitions are highly fragmented and lacking in pattern [44,45,47]. Relative Amplitude (RA) has also frequently been used as a non-parametric approximation of circadian robustness and is calculated as a ratio of the difference between the most active 10 h of the day (M10) and the least active 5 h (L5) divided by their sum. For this index, higher values indicate more differentiation between M10 and L5 [47,48].

### 2.3. Behavioral Data

Parent-reported questionnaire data were obtained for 44 of the participants with DS and 30 participants with TD using the Behavior Rating Inventory of Executive Function–Parent Report (BRIEF-2) survey [49] and the Children’s Sleep Habits Questionnaire (CSHQ) parent form [50]. Young adults with TD did not complete the questionnaires, and incomplete questionnaire data were obtained for 14 participants with DS and 4 with TD. The BRIEF-2 measures executive function via parent observation of children’s behavior on 63 response items measured on a 3-point Likert scale. It provides a general executive score as well as domain scores in 12 areas. The BRIEF-2 has been frequently used in studies of children with DS [1,51]. The CSHQ has been used to estimate sleep behavior in children with DS and other forms of intellectual disability [3,52] and has demonstrated an ability to identify behavioral sleep problems in children with DS aged 6–17 years [6]. The form contains 33 3-point Likert scale questions that provide a general score as well as domain scores in the areas of bedtime resistance, sleep onset delay, sleep duration, sleep anxiety, night waking, parasomnias, sleep-disordered breathing and daytime sleepiness. Higher scores indicate better quality sleep behavior.

### 2.4. Cognitive Data

Cognitive data for participants with DS were gathered via laboratory assessment using the KBIT-II and a novel touchscreen battery, the Arizona Memory Assessment for Preschoolers and Special Populations (A-MAP^TM^) [53]. These data were not available for most participants with TD, so the current study focused on reporting cognitive data for the groups with DS. The KBIT-II is designed to concisely assess verbal and nonverbal knowledge via three subtests (verbal, matrices, and riddles). Intended for cultural fairness in item selection and norming procedures, the combined subtest scores yield a comprehensive standard IQ score. The present study used the KBIT-II raw verbal score to estimate verbal IQ. The KBIT-II is appropriate for ages 4–90 and has a precedence for measuring cognition in studies of individuals with DS [14,39]. The A-MAP^TM^ is a multi-domain memory assessment, designed for young children and populations with intellectual and developmental disabilities, delivered via tablet. Of particular interest in this study were the A-MAP^TM^ tasks measuring immediate verbal recall, immediate object recognition, scene memory, object-context binding, and reaction time on a complex executive function task; all domains found to be affected in previous studies of circadian disruption [19,27].

58 participants with DS completed part or all of the A-MAP^TM^ under laboratory conditions between 2017 and 2020. In the immediate visual recall task, participants were shown and had the opportunity to interact with 12 target images and were then asked to point to each of these 12 images when they were presented along with two distractors. Possible scores ranged from 0 to 12 on this measure. In the verbal recall task, participants were asked to list 12 objects they had just viewed, interacted with, and named. Possible scores were 0 to 36 on the verbal recall task. In the spatial recall task, participants were first shown the contents of a set of boxes, which each contained either nothing or an object such as a toy marble. Afterward, they were asked to touch the boxes which had contained the toys. Participants were given six trials to correctly place all toys shown for each trial. Possible scores were 0 to 24. In the scene recall task, participants were first shown a series of 12 scene images. They were then asked to select each image they saw when it was presented with two distractor scenes. Possible scores for the scene recall task were 0 to 12. Also included was a task measuring item-to-context binding, in which participants were shown several items that were hiding in boxes, then were asked after all boxes were closed to place the items in the correct boxes. Possible scores for this measure were 0 to 24. In the executive function task, participants were first introduced to an animal and that animal’s preferred object (i.e., in Form A, a mouse who like cheese). They learned how to select all the cheese and feed it to the mouse. They were then introduced to an alternate item (a distractor) that the first animal doesn’t like (i.e., a beach ball for Form A), and were told to select only the items that the mouse likes on each trial. Following that, they were introduced to the seal, who only likes beach balls. The portion of the task used in this study was the Alternate portion, in which either cheese or a beach ball was displayed in a central box, either with or without distractors surrounding the box, and participants were told to select the animal that likes the item in the box as quickly as possible. They were timed on 25 trials, following which the trial times were averaged for each participant. 

### 2.5. Statistical Analyses

All analyses were performed in R Studio version 1.3.1073. To assess group differences across the sleep, phase, and circadian markers with various group sizes, we performed permutation ANOVAs (Kruskal-Wallace tests for the variables with non-normally distributed residuals), followed by Holm correction for multiple comparisons. To assess group differences on the CSHQ, permutation ANOVAs were also used; Kruskal-Wallace tests for the variables with non-normal residuals. 

In order to explore potential cognitive outcomes related to phase and circadian rhythmicity, we constructed a set of hierarchical linear models with terms for the independent variable IS and covariates SE, age, gender, and the number of full weekend days of actigraphy collected. We assessed as outcome measures verbal intelligence, executive function task timing, immediate verbal recall, immediate spatial recall, immediate scene recall, immediate visual recall, and object-context binding. Two outcome measures from the A-MAP^TM^ assessment (verbal recall and object-context binding) were logarithmically transformed and another (visual recall) was square root transformed to improve their normalcy. All other assumptions for linear modeling were met. Outliers of more than 3 standard deviations from the mean were excluded. In the forward stepwise modeling phase, main effects were entered in the first step. In the second step, the full models included an interaction term for IS × age (measured continuously), but this interaction term was then trimmed from the model in the final step if it had a *p*-value greater than 0.10 and did not contribute significantly to the model multiple *R*^2^. We followed the ANOVAs and linear models with Holm correction for multiple comparison. All *p* values reported in the Results are Holm-corrected values.

## 3. Results

### 3.1. Sleep and Phase Timing

The children, adolescents, and adults with DS slept on average nearly an identical total amount as their counterparts with TD (TST for group *p* > 0.05). Children with DS slept on average only 13 mins less than children with TD, and adolescents with DS slept on average only 1 min less than adolescents with TD. For all ages with DS, sleep efficiency (SE) was significantly lower and wake after sleep onset (WASO) was significantly higher (group *p* < 0.001 for both). Significant age differences were also noted for WASO (*p* < 0.05), where children in both groups had the highest WASO scores (most time awake after sleep onset) and adolescents in both groups had lower WASO than their childhood counterparts. Adults with TD scored lowest on this measure while adults with DS had slightly higher WASO than did adolescents with DS. Results for sleep measures are summarized in Table 2.

**Figure 2:**
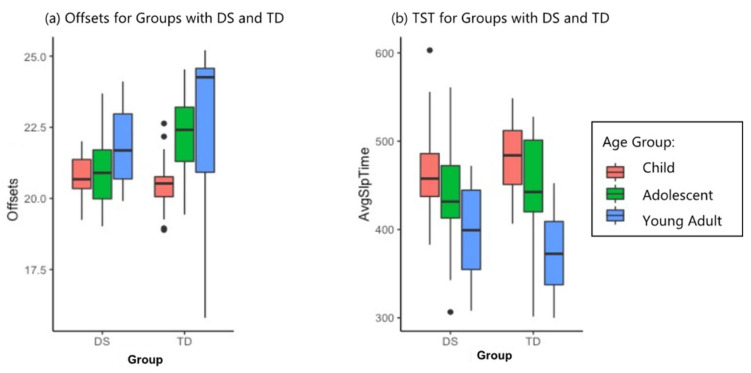
Phase timing and average total sleep time estimates. (**a**) Average offsets (time of falling asleep) by group and age. The adolescent phase shift at the onset of puberty is visible in the TD group, where the mean offset was 1 h 45 min later for adolescents than children. Adolescents with DS, on the left, fell asleep only 15 min later than children with DS. (**b**) Total sleep time (TST) by group and age, illustrating the similar amounts of TST between the two groups at each age.

In terms of phase timing (average time of day of waking, falling asleep, and the most active period), there were no significant differences between the DS and TD groups for children aged 6–10 in onset, offset, and acrophase values. However, the adolescents with TD demonstrated a phase shift to an average offset or bedtime of 1 h 45 min later than the children with TD, while the adolescents with DS did not demonstrate a comparable phase shift, falling asleep only 15 min later than children with DS. Despite these measured differences, the ANOVA for offsets was significant for age only (*p* < 0.01). Onset and acrophase ANOVAs, however, yielded significant results for both age (*p* < 0.05, *p* < 0.001, respectively) and group (*p* < 0.01 for both). The offset and TST results can be seen in Figure 2; the permutation ANOVA results are summarized for phase variables in Table 2. 

### 3.2. Stability and Robustness of Circadian Markers

**Figure 3:**
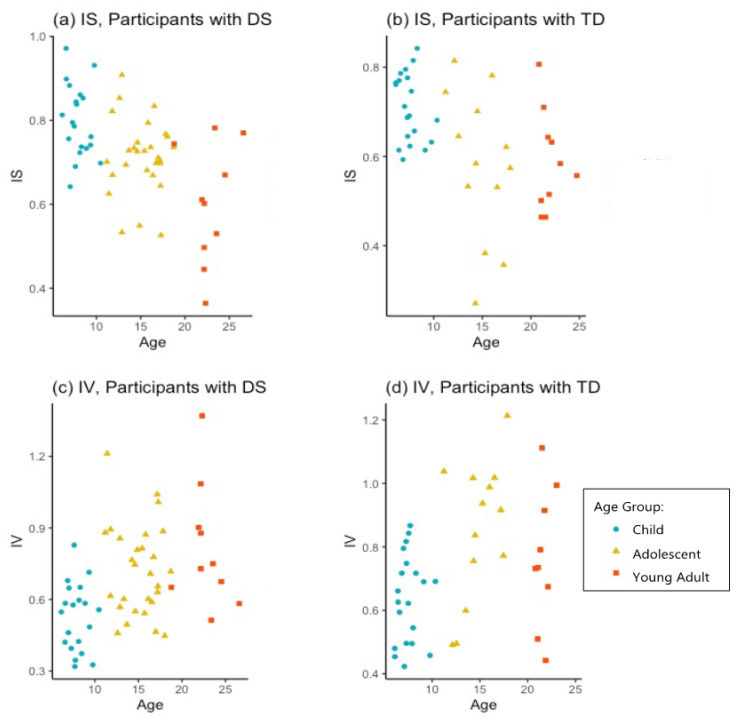
IS and IV across ages. (**a**) IS for individuals with DS declined from childhood through young adulthood, indicating less stability. (**b**) IS for individuals with TD declined for adolescents but did not decline further for young adults. (**c**) IV increased in individuals with DS, indicating more randomness in within-day sleep-wake transitions, from childhood through adulthood. (**d**) IV in individuals with TD was highest for adolescents, but less random for young adults.

Stability of sleep-wake rhythms or interdaily stability (IS) was strongest for children with DS (0.80) followed by children with TD (0.71). In both groups, IS was significantly worse for adolescents (0.71 for those with DS; 0.58 for TD) compared to children (age *p* < 0.001; group *p* < 0.05). The adults with DS demonstrated a further drop in stability (0.60) compared to adolescents with DS, while the adults with TD held steady (0.59) compared to adolescents with TD. Intradaily variability (IV) also showed significant age effects (*p* < 0.001). For this measure, a higher value represents more frequent sleep-wake transitions and a worse score. Similar to IS, IV was also most stable for children with DS (0.53) followed by children with TD (0.64). Adolescents in both groups demonstrated higher (more fragmented) IV than children; (0.72) for adolescents with DS and (0.85) for adolescents with TD. Adults with DS showed still more fragmented IV (0.81) than adolescents, while adults with TD (0.77) were less variable than adolescents with TD on this measure. The IS and IV values for all participants with DS across ages, sorted by age group, can be seen in Figure 3.

**Figure 4:**
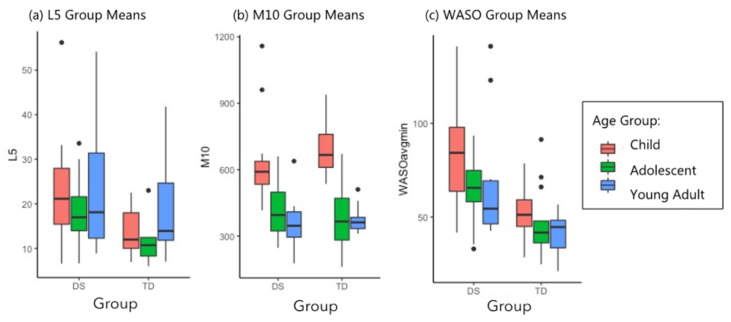
Group means by age for L5, M10, and WASO. (**a**) The L5, or the sequence of least-active 5 h in the 24-h period was higher for participants with DS (*p* < 0.01). (**b**) There were no significant group differences for M10, or the most active 10-h sequence during the 24-h period (*p* > 0.05). (**c**) The WASO ANOVA indicated significantly more minutes awake after sleep onset for participants with DS than for participants with TD (*p* < 0.001).

A group effect and an age effect (*p* < 0.05 and *p* < 0.001, respectively) were noted for the FFT of the 24-h sleep-wake cycles. In both groups, children demonstrated the highest amplitude to their daily activity rhythms as measured by the FFT (0.31 for children with DS; 0.27 for children with TD). Adolescents in both groups had significantly lower FFT (0.22 for adolescents with DS; 0.14 for adolescents with TD) and adults had very similar FFT values relative to adolescents in both groups (0.23 for adults with DS; 0.15 for adults with TD). We hypothesized that the higher amplitude values for participants with DS might be driven by their more frequent nighttime awakenings and concomitant nighttime restlessness rather than by daytime activity levels. Therefore, we examined group differences between L5 and M10 to understand the mean hourly activity level during the sequence of the least active 5 h (L5) of the night and the mean hourly activity level during the sequence of the most active 10 h (M10) of the day. Participants with DS had higher L5 levels than counterparts with TD across all 3 age groups, indicating they were more active at night than controls (group *p* < 0.05). In terms of M10, participants with DS had activity levels that were not significantly different than participants with TD (group *p* > 0.05), indicating that on average, the two groups had similar amounts of daytime activity. As detailed in Table 2, we also found higher WASO for participants with DS: 56% higher for children with DS than children with TD, 38% higher for adolescents with DS, and 67% higher for young adults with DS (group *p* < 0.001). The nighttime measures of WASO and L5 were higher for participants with DS, while the daytime activity measure of M10 was not significantly elevated for participants with DS. The group means by age for L5, M10, and WASO can be seen in Figure 4.

### 3.3. Parent-Reported Sleep Data

The ANOVAs for the parent reported CSHQ indicated significant main effects of age for bedtime resistance (*p* < 0.01), sleep duration (*p* < 0.001), and nighttime wakings (*p* < 0.01). Significant main effects for group were indicated for bedtime resistance (*p* < 0.05), sleep-disordered breathing (*p* < 0.01), and the CSHQ total score (*p* < 0.05). The most nighttime wakings on average were reported for children with DS (at 5.2). Finally, for the sleep-disordered breathing subscale, the highest average scores were seen for children with DS (4.7) and adolescents (4.64) with DS. Complete results for the CSQH ANOVAs are displayed in Table 3. 

### 3.4. Cognitive Outcomes in Participants with DS

**Figure 5:**
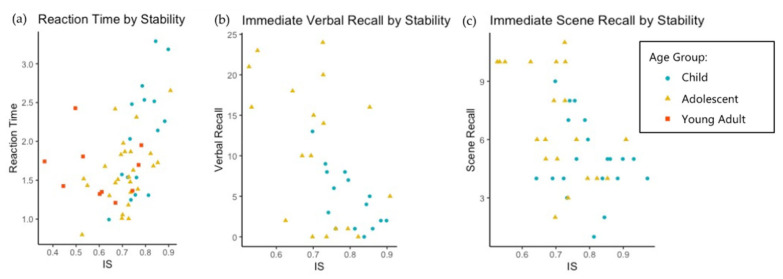
Interdaily stability and cognitive measures for participants with DS. (**a**) Reaction time was positively correlated with IS, but examining the relationship by age, there is preliminary evidence of a different relationship for adults (red squares). (**b**) Immediate verbal recall had a negative correlation with IS. (**c**) Immediate scene recall had a negative correlation with IS.

Results of the regression models are summarized in Table 4 and Figure 5.

52 individuals with DS completed the KBIT-II verbal scale. Neither IS nor SE nor any covariates were significant predictors of verbal KBIT-II performance, all *p* > 0.05. For the executive function task, 41 individuals with DS participated fully. Given that only 3 individuals were below chance performance (50%) on correctly distinguishing the correct object in this task, we covaried with performance on the task. Two outliers were excluded according to the previously stated guideline, bringing the total *n* of this linear model to 39. IS and age both positively correlated with reaction time (*p* < 0.05 for both); (see Table 4 and Figure 5). Sleep efficiency was not a significant predictor, *p* > 0.05. The model explained about 50% of the variation in this measure. For general executive function (BRIEF-2 GEC), 40 participants completed the measure; there were no significant results; all *p* > 0.05.

For immediate verbal recall, 27 children and adolescents with DS completed the task. The linear model indicated IS alone was significantly correlated (*p* < 0.05), and the correlation was negative. Sleep efficiency was not a significant predictor, *p* > 0.05. The model accounted for about 39% of the variation in immediate verbal recall. Immediate scene recall was completed by 40 children and adolescents with DS. One outlier was excluded, leaving the final *n* at 39. Sleep efficiency was not a significant predictor, *p* > 0.05. For immediate scene recall, a negative correlation was found with interdaily stability (*p* < 0.05), and the model accounted for about 39% of variability on this measure. The object-context binding task was completed by 54 children and adolescents with DS. Object-context binding was significantly correlated with age (*p* < 0.01) but not with IS or SE (*p* > 0.05 for both). The model accounted for about 42% of variation on this measure. For immediate visual and spatial recall, none of the tested variables predicted performance; all *p* > 0.05. 

## 4. Discussion

The primary goals of the current study were to report on sleep, phase timing, and circadian robustness markers demonstrated by children, adolescents, and young adults with DS compared to peers with TD; and to assess for correlations with cognitive and executive function measures that have been noted previously in the TD literature.

### 4.1. Sleep and Phase Timing

Although it was anticipated that SE and WASO would show marked group differences, TST was unexpectedly similar across groups. Children in both groups slept substantially less than the recommended 9–12 h per 24-h period, and adolescents in both groups slept less than the 8–10 h recommended [54]. The current study did not document nighttime technology use by participants (i.e., cell phones, tablets) that may have prolonged sleep onset latency and contributed to lower TST. Despite the similar TST, a phase timing gap between the two groups became evident by the age of 12 years. This phase timing gap occurred when the adolescents with TD in this study shifted to a 1 h, 45 min later average bedtime, while adolescents with DS, measured with the same equipment and methods, showed no phase shift. This phase timing gap diminished by the end of the twenties but did not disappear entirely. In considering why the adolescents with DS did not phase shift, one explanation may be that they have such a high degree of sleep pressure (built up from previous nights of fragmented sleep) that an early offset becomes for them a biological imperative. A second possibility could be that adolescents (and even adults) with DS have more strictly parentally regulated schedules than do counterparts with TD after childhood, and this serves to perpetuate the earlier bedtimes of the childhood years. Considering this possibility, we examined the CSHQ subscale of Bedtime Resistance. If adolescents with DS are held to a strict bedtime schedule, we might expect to see higher parent-reported bedtime resistance scores for them. Parents of participants with DS did report substantially more resistance from adolescents than children, and even more resistance from young adults with DS. Parents of participants with TD, on the other hand, reported less bedtime resistance with adolescents than with children. The CSHQ bedtime resistance ANOVA was significant for group differences, *p* < 0.05 (see Table 3). It may be noted that youth and young adults with DS could also differ from their counterparts with TD in the degree to which they experience other psychosocial factors, i.e., late-night schoolwork and after-school jobs and in the degree to which these factors might influence their phase timing. However, differences in psychosocial factors, such as parental regulation, schoolwork and job loads, may offer only a partial explanation. It is likely that a combination of these factors with high sleep pressure and other possible unknown factors may together account for the earlier offsets seen in adolescents with DS in this study. Future research could employ an experimental design to test hypotheses about the lack of a phase shift in adolescents with DS. 

### 4.2. Stability and Robustness of Circadian Markers

As expected, children with DS demonstrated robust sleep-wake rhythms, corroborating previous findings regarding the establishment of healthy circadian rhythms in the early years in this population [13]. We predicted that sleep-wake rhythms would become less stable in the adolescent years and that for at least a subset of young adults with DS, lower stability would then persist in adulthood. The current study found support for these hypotheses in both IS and IV when examined across the three age groups with DS. IS declined from a very strong 0.80 average in the childhood years to about 0.60 in younger adults in an apparently linear trajectory that did not appear to slow or reverse by the age of 26. IV values indicate greater variability in sleep-wake rhythms at each successive timepoint for people with DS, from a 0.53 average in childhood, to 0.72 in adolescence, to 0.81 in young adulthood, also in an apparent linear trajectory. Young adult participants with TD, in contrast, did on average hold steady or begin to recover from adolescent lows on both IS and IV by age 26. The results of the current study indicate that IS and IV may look different for adults with DS than adults with TD. Future research could measure IS and IV in adults with DS longitudinally to examine whether IS and IV are correlated with cognitive performance as adults age.

A result noted in the mouse research [37,55] and replicated in the current study was the elevated amplitude and FFT of activity in the 24-h sleep-wake cycle for participants with DS. The current study was able to add an examination of the nonparametric measures L5 (the least active 5-h sequence within a 24-h period, generally at night) and M10 (most active 10-h sequence, generally during the day) by group to provide more information about whether the elevated amplitude for individuals with DS might be driven by daytime or nighttime activity. Our statistical analysis indicated that the elevated amplitude and FFT in individuals with DS was likely due to nighttime activity, which was higher for participants with DS than for controls as measured by L5 (*p* < 0.05). This can be seen also in Figure 1c,d, where higher nighttime activity spikes are visible for adolescents with DS. Daytime activity counts, as measured by the M10, were non-significantly different between groups (*p* > 0.05), which indicates that it is likely not daytime activity that is driving the group difference. Additionally, measurement of WASO was included in the current study and was examined when looking for the source of the elevated FFT found for participants with DS. The current study did find higher WASO for participants with DS, indicating more nighttime wakings, which are likely to be accompanied by greater nighttime movement: 56% higher for children with DS than children with TD, 38% higher for adolescents with DS (group *p* < 0.001). The higher nighttime measures of WASO and L5 for participants with DS demonstrate that nighttime activity was responsible for the elevated FFT values for participants with DS. However, future studies using more objective sleep measurements could provide information helpful to the development of sleep treatment plans for individuals with DS as well as with other intellectual and developmental disabilities.

### 4.3. Parent-Reported Sleep Health

Overall, the CSHQ mirrored some of the results of actigraphy, but suggested there are significant gaps in the information acquired by the two measures. The CSHQ bedtime resistance subscale differences for both group and age indicate difficulty at bedtime as individuals with DS age (see Table 3). Subscale scores for sleep duration were worse for both groups of adolescents than for their childhood counterparts with the highest ratings for this domain in adolescents and adults with DS, indicating parents are aware of sub-optimal sleep length for these adolescents and adults with DS. However, parents of children and adolescents with TD and of children with DS did not indicate sleep duration as a problem, even though their children also slept on average 1–2 h less than the low end of the recommended range for their age groups as measured with actigraphy. A second domain for which the CSHQ did not align with the actigraphy results was that of night wakings. The CSHQ night wakings domain failed to find group differences that would mirror the WASO group differences found via actigraphy in which children, adolescents and adults with DS spent more time awake after the onset of sleep *(p* < 0.001). Sleep duration, as well as night wakings, sleep onset delay, and sleep-disordered breathing may be phenomena that are more difficult to measure accurately by parent report during the adolescent years, when children are more independent in their own rooms and may sleep and wake differently than parents realize. Similar conclusions were drawn in a recent study of CSHQ construct validity that found no correlation between the above-mentioned domains and PSG measurement [56]. This underscores the importance of objective sleep measurement for these phenomena at this age.

### 4.4. Executive Function Measures

Contrary to our hypothesis, IS had a positive correlation with reaction time for individuals with DS. Why the correlation was positive rather than negative may be related to cognitive control—the ability to behave in a goal-directed manner in response to external and internal demands. Proactive cognitive control is the mechanism through which people retain information in working memory and prepare for any necessary actions toward reaching current goals while blocking interference from external and internal sources [57]. Maintaining proactive control may require substantial cognitive resources in youth with DS and hence could slow down reaction time considerably. Switching tasks such as the one utilized in the current study require cognitive control flexible enough to make adjustments that quickly adapt a person to changing task conditions. This seems to be achieved through network activation involving frontal theta oscillations, particularly in the Anterior Cingulate Cortex (ACC). Several studies of neurotypical adults have reported on the relationship between slowed reaction time in task-switching and increased medial PFC (mPFC) theta rhythms [58,59,60]. The ACC has been indicated as a highly atypical brain region in individuals with DS [16], which may contribute to difficulty engaging proactive cognitive control. In the current study, it may be that individuals with DS who exhibited more stable sleep rhythms were better able to activate cognitive control networks engaging the mPFC, albeit with a cost of longer reaction time. For young individuals with DS, a stronger IS may have specifically conferred the ability to better direct attention to the task at hand and sustain goal-directed motivation in the face of new information. EEG measurement was not included in the current study but could be a key component of future research aimed at understanding neural activity associated with cognition in children, adolescents and adults with DS and sleep effects on these processes. As such, future research concerned with executive function in individuals with DS may wish to record EEG during a similarly complex reaction time task to assess frontal theta oscillations in this population.

### 4.5. Memory Measures

In the current study, linear models suggested that stronger IS was associated with worse performance on immediate recall of verbal and scene prompts, but was not related to spatial recall, visual recall, or object-context binding on the novel A-MAP^TM^ assessment. Adolescents remembered on average more of the verbal and scene stimuli but had less stability in their sleep-wake rhythms than did children. Previous findings in studies of neurotypical individuals found that verbal memory was specifically associated with circadian disruption [19]. However, while that study found that better sleep-wake rhythms were related to better cognitive and behavioral performance, the current study found the opposite. 

The current study only included children and adolescents with DS in the memory models while Wright et al. [19] studied neurotypical adults, and it is a distinct possibility that developmental processes are mediating the interaction between IS and memory until the end of brain development, in early adulthood. As similarly concluded by Astill et al. in a meta-analysis of neurotypical children [61], sleep and cognition, methodological issues and brain development immaturities may be behind the different sleep rhythm-cognitive correlations found in this study and in previous studies of neurotypical adults. However, it is still not clear why in our sample, IS was negatively correlated with verbal and scene memory.

Previous research into sleep-dependent memory, focused on children with DS, has also yielded results that differ from those found in research with neurotypical children. Specifically, Spano et al. [62] found that young children with DS were unable to benefit from napping in list learning performance, while neurotypical children did. The current study may perhaps provide further indication that children with DS can be differentially affected by sleep in middle childhood and adolescence. Future work in sleep and cognition in individuals with DS may wish to consider sleep stages in research design, as disruption at different stages may correlate with different memory outcomes.

Limitations of the current study include those imposed by the methodology of actigraphy for sleep, phase and circadian robustness estimation; the small group sizes for young adults; the absence of cognitive data for participants with TD; and the smaller quantity of cognitive data collected for young adults with DS. Specifically, verbal and scene recall assessments were not completed by this group. It is possible that the young adult group would conform to the same pattern noted for children and adolescents with DS of negative correlation between stability and verbal or scene memory, but it is also possible that they could show a more positive correlation, as was predicted. More work is needed to understand the nature of circadian rhythms in adolescents and adults with DS and their relationship with cognitive function. It should be noted that none of the cognitive outcomes in this age-range (KBIT-II, EF, or memory) demonstrated relations with sleep efficiency suggesting that this age range may not demonstrate the correlations that were previously measured in preschool groups [1,63].

## 5. Conclusions

It cannot be overemphasized that children and adolescents with both DS and TD slept on average substantially less than current recommendations. This indicates the presence of a trend toward insufficient childhood sleep, noted previously by Astill et al. [61], that cannot be seen as a positive direction for their health and safety. Although children and adolescents with DS slept the same amount as counterparts with TD in the current study, they maintained a distinct tendency toward earlier, or “lark” phase timing and did not demonstrate the typical teenage phase shift. This finding may be due to a combination of factors which could be examined in future research, including the impact of sleep pressure. This study was able to add specificity to previous findings of elevated amplitude of activity cycles in individuals with DS by separately considering daytime versus nighttime activity, and demonstrating that nighttime activity (i.e., movement during the primary sleep period, at night) was the element driving the elevated amplitude. This finding further underscores the need for sleep treatment for individuals with DS aimed at identifying and mitigating causes of restlessness throughout the night. The current study also found preliminary evidence of what could be a failure of sleep rhythm stability and consolidation to recover strength after the adolescent years for individuals with DS. This calls for further investigation in light of the connection observed between circadian rhythm disruption and the development of Alzheimer’s dementia in the general population, as noted previously i.e., [30,31,32,33]. Finally, more work is needed in assessing sleep-wake rhythms and their cognitive correlations across the lifespan in individuals with DS, as our study suggests developmental change in these processes. In such work, priority must be given to the most exact measurement methods possible for recording sleep, sleep rhythms, and potentially, brain activity during cognitive testing. 

## Figures and Tables

**Table 1:** Demographic Information.

Age, Group	Total (Females)	Mean Ages (Mean Female Ages)
Children with DS	20 (8)	7.95 ± 1.16 (7.75 ± 1.32)
Children, TD	20 (10)	7.53 ± 1.15 (7.27 ± 0.84)
Teenagers with DS	28 (16)	15.11 ± 2.21 (15.59 ± 2.02)
Teenagers, TD	13 (5)	14.84 ± 2.12 (16.51 ± 1.74)
Adults with DS	10 (3)	22.76 ± 2.02 (24.42 ± 2.22)
Adults, TD	10 (6)	21.94 ± 1.17 (22.07 ± 1.38)

**Table 2:** ANOVA results for sleep, phase, and circadian markers; M (SD) grouped by individuals with DS and with TD over 3 age groups: children 6–10, adolescents 11–18, and young adults 19–26.

Measures	Children, DS *n* = 20	Children, TD *n* = 20	Adolescents, DS *n* = 28	Adolescents, TD *n* = 13	Adults, DS *n* = 10	Adults, TD *n* = 10	F, Age F, Group F, Interaction	*p*, Age *p*, Group *p*, Interaction	*p*, Corrected, Age *p*, Corrected, Group *p*, Corrected, Interaction
TST	469.37 (51.59)	482.40 (40.43)	438.32 (53.40)	439.61 (66.73)	398.61 (55.83)	376.12 (51.49)	F = 19.02 F = 0.81 F = 0.47	1.13 × 10^−7^ *** ns *ns*	2.71 × 10^−6^ *** ns *ns*
SE	83.85 (5.05)	89.32 (2.68)	85.98 (3.37)	89.63 (2.62)	83.73 (6.67)	87.36 (3.52)	F = 2.19 F = 24.72 F=0.60	*ns* 2.94 × 10^−6^ *** ns	*ns* 5.58 × 10^−5^ *** ns
WASO	83.18 (24.93)	53.23 (13.01)	64.74 (16.80)	46.86 (18.91)	69.11 (34.71)	41.27 (10.96)	F = 5.81 F = 38.13 F = 0.25	4.17 × 10^−3^ ** 1.63 × 10^−8^ *** ns	0.03 * 4.07 × 10^−7^ *** ns
Onsets	6.69 (0.60)	7.014 (1.140)	6.59 (1.08)	7.57 (1.21)	7.19 (1.24)	7.80 (2.97)	F = 5.92 F = 14.92 F = 2.11	3.8 × 10^−3^ ** 2.1 × 10^−4^ *** ns	0.03 * 3.32 × 10^−3^ ** ns
Offsets	20.76 (0.77)	20.48 (0.96)	20.92 (1.21)	22.23 (1.59)	21.83 (1.47)	22.40 (3.53)	*χ*^2^ = 16.03 *χ*^2^ = 2.00	3.3 × 10^−4^ *** ns	4.95 × 10^−3^ ** ns
Acrophase	13.62 (0.87)	13.59 (0.83)	14.16 (1.04)	14.88 (1.54)	14.30 (1.30)	15.82 (1.80)	F = 16.74 F = 14.04 F = 5.23	5.57 × 10^−7^ *** 3.3 × 10^−4^ *** 5.82 × 10^−3^ **	1.23 × 10^−5^ *** 4.95 × 10^−3^ ** 0.03 *
IS	0.80 (0.09)	0.71 (0.08)	0.71 (0.09)	0.58 (0.17)	0.60 (0.14)	0.59 (0.11)	F = 15.71 F = 9.53 F = 2.30	1.32 × 10^−6^ *** 2.66 × 10^−3^ ** ns	2.78 × 10^−5^ *** 2.66 × 10^−2^ * ns
IV	0.53 (0.141)	0.64 (0.14)	0.72 (0.19)	0.85 (0.22)	0.81 (0.26)	0.77 (0.22)	F = 14.90 F = 3.41 F = 0.96	2.44 × 10^−6^ *** ns *ns*	4.89 × 10^−5^ *** ns *ns*
FFT	0.31 (0.07)	0.27 (0.06)	0.22 (0.06)	0.14 (0.06)	0.23 (0.17)	0.15 (0.040)	*χ*^2^ = 29.10 *χ*^2^ = 6.52	4.79 × 10^−7^ *** 0.01069	1.10 × 10^−5^ *** 0.034 * ns
L5	22.53 (10.93)	13.76 (5.02)	17.64 (6.08)	14.94 (14.89)	23.00 (15.30)	18.53 (10.72)	F = 1.90 F = 8.26 F = 0.75	*ns* 5.01 × 10^−3^ ** ns	*ns* 0.034 * ns
M10	614.73 (171.56)	687.0 (107.28)	403.75 (110.36)	376.74 (138.78)	363.89 (122.82)	376.69 (62.81)	F = 49.83 F = 0.42 F = 1.88	1.77 × 10^−15^ *** ns *ns*	4.59 × 10^−14^ *** ns *ns*

* = significant at *p* = 0.05 level; ** = significant at *p* = 0.01 level; *** = significant at *p* = 0.001 level; *ns* = not significant.

**Table 3:** ANOVA results for CSHQ domains; individuals with DS and with TD over 3 age groups: children 6–10, adolescents 11–18, and young adults 19–26.

CSHQ Measures	Children, DS *n* = 20	Children, TD *n* = 20	Teens, DS *n* = 28	Teens, TD *n* = 13	Adults, DS *n* = 10	*χ*^2^ or F, Age *χ*^2^ or F, Group	*p*, Age *p*, Group	*p*, Corrected, Age *p*, Corrected, Group
Bedtime Resistance	8.05 (3.52)	7.35 (2.41)	8.96 (3.72)	6.25 (0.62)	13 (1.32)	*χ*^2^ = 15.75 *χ*^2^ = 9.36	3.80 × 10^−4^ 2.21 × 10^−3^	4.95 × 10^−3^ ** 0.02 *
Sleep Onset Delay	1.25 (0.64)	1.2 (0.41)	1.32 (0.7)	1.42 (0.52)	1.11 (0.33)	*χ*^2^ = 1.76 *χ*^2^ = 0.89	*ns* *ns*	*ns* *ns*
Sleep Duration	4.2 (1.51)	3.7 (1.42)	4.79 (1.83)	4.08 (1.8)	7 (0.5)	*χ*^2^ = 19.64 *χ*^2^ = 7.09	5.45 × 10^−5^ 7.75 × 10^−3^	9.81 × 10^−4^ *** 0.03 *
Sleep Anxiety	5.75 (1.89)	5.15 (2.01)	5.61 (1.99)	4 (0.43)	4.67 (0.71)	*χ*^2^ = 1.1 *χ*^2^ = 7.18	*ns* 7.39 × 10^−3^	*ns* 0.03 *
Night Wakings	5.2 (2.12)	4.05 (1.15)	4.07 (1.63)	3.54 (0.66)	3.22 (0.67)	*χ*^2^ = 10.78 *χ*^2^ = 0.004	4.57 × 10^−3^ *ns*	0.03 * ns
Parasomnias	9.2 (2.19)	8.75 (1.94)	9.11 (2.27)	8.08 (1.38)	7.67 (1.00)	F = 2.24 F = 2.01	*ns* *ns*	*ns* *ns*
Sleep Disordered Breathing	4.7 (2.23)	3.25 (0.55)	4.64 (2.57)	3.08 (0.28)	3.78 (1.09)	*χ*^2^ = 0.16 *χ*^2^ = 14.40	*ns* 1.48 × 10^−4^	*ns* 2.52 × 10^−3^ **
Daytime Sleepiness	12.8 (7.76)	11.3 (2.87)	13.36 (2.83)	11.58 (2.81)	13.11 (1.76)	*χ*^2^ = 6.83 *χ*^2^ = 4.88	3.29 × 10^−2^ 2.72 × 10^−2^	*ns* *ns*
CSHQ Total	46.15 (6.54)	42.15 (7.37)	13.36 (2.83)	40.54 (4.68)	48.22 (2.99)	F = 0.14 F = 10.91	*ns* 1.41 × 10^−3^	*ns* 0.02 *

* = significant at *p* = 0.05 level; ** = significant at *p* = 0.01 level; *** = significant at *p* = 0.001 level; *ns* = not significant.

**Table 4:** Linear Model results for executive function, memory, and KBIT-II verbal intelligence models for individuals with DS.

Executive, Cognitive Outcomes	Model F Statistic	Degrees of Freedom	Interdaily Stability (IS) *p* Value	Sleep Efficiency (SE) *p* Value	Age *p* Value	Gender *p* Value	IS × Age Interaction *p* Value	Model Multiple *R*^2^	Significant *p* Values, Corrected
Reaction Time	5.363	6.32	0.00278 **	0.693734	0.03137 *	0.808016	0.02159 *	0.5014	IS: 0.011 * Age: 0.043 * Is × Age: *ns*
BRIEF	0.198	6.45	0.3658	0.7704	0.5104	0.7697	0.487	0.02152	*ns*
Verbal Recall	4.557	4.28	0.00274 **	0.22320	0.95120	0.59583	*na*	0.3943	IS: 0.014 *
Object-Context Binding	6.939	5.48	0.22554	0.48540	0.00112 **	0.03389 *	*na*	0.4195	Age 6.72 × 10^−3^ **
Spatial Recall	5.228	5.32	0.1266	0.6160	0.0272 *	0.5816	*na*	0.4496	*ns*
Scene Recall	3.142	5.33	0.0078 **	0.2005	0.9070	0.3846	*na*	0.3225	IS: 0.03 *
Visual Recall	3.868	5.51	0.1183	0.6864	0.0616	0.6172	0.101	0.275	*ns*
KBIT-II Verbal	0.138	4.52	0.542	0.713	0.992	0.783	*na*	0.01697	*ns*

* = significant at *p* = 0.05 level; ** = significant at *p* = 0.01 level; *ns* = not significant.

## Data Availability

The data and code are available at https://github.com/MDD-UA/Sleep-Wake-Rhythms.git (accessed on 19 August 2021).
